# Data exploration on the factors associated with cost overrun on social housing projects in Trinidad and Tobago

**DOI:** 10.1016/j.dib.2023.109966

**Published:** 2023-12-15

**Authors:** Aaron Anil Chadee, Chamari Allis, Upaka Rathnayake, Hector Martin, Hazi M Azamathulla

**Affiliations:** a Department of Civil and Environmental Engineering, The Faculty of Engineering, The University of West Indies, St. Augustine 32080, Trinidad and Tobago; b Department of Quantity Surveying, Faculty of Engineering, Sri Lanka Institute of Information Technology, Malabe 10115, Sri Lanka; c Department of Civil Engineering and Construction, Faculty of Engineering and Design, Atlantic Technological University, Ash Lane, F91 YW50 Sligo, Ireland; d Department of Civil Engineering, Queen's University Belfast, University Belfast, United Kingdom

**Keywords:** Project management, Construction industry, Root causes, Political, Technical, Cost overruns, Small Island developing states

## Abstract

This data article explores the factors that contribute to cost overrun on public sector projects within Trinidad and Tobago. The data was obtained through literature research, and structured questionnaires, designed using open-ended questions and the Likert scale. The responses were gathered from project actors and decision-makers within the public and private construction industry, mainly, project managers, contractors, engineers, architects, and consultants. The dataset was analysed using frequency, simple percentage, mean, risk impact, and fuzzy logic via the fuzzy synthetic evaluation method (FSE). The significance of the analysed data is to determine the critical root causes of cost overrun which affect public sector infrastructure development projects (PSIDPs), from being completed on time and within budget. The dataset is most useful to project and construction management professionals and academia, to provide additional insight into the understanding of the leading factors associated with cost overrun and the critical group in which they occur (political factors). Such understanding can encourage greater decisions under uncertainty and complexity, thus accounting for and reducing cost overrun on public sector projects.

Specifications Table

**Table:** 

Subject	Engineering
Specific subject area	Construction Cost Management under the Project Management
Data format	Raw Analysed
Type of data	Table
Data collection	Literature research and structured survey questionnaire both hard and soft copy (SurveyMonkey) Forty-one (41) factors related to cost overrun on construction projects were extracted from 37 journals, through literature research. Structured questionnaires designed with both open-ended questions and the 7-point Likert scale captured the demographic data and the views of the respondents. Survey questionnaires were distributed to construction professionals such as Project Managers, Contractors, Engineers, Architects, and Consultants. A total of 84 responses were obtained through the online SurveyMonkey application and 66 via hard copy. A total of 150 responses were obtained and were used in data generation. The data was analysed with the aid of software such as Statistical Package for Social Sciences (SPSS) IBM 25 and Microsoft Excel 2018.
Data source location	Primary data sources: University of the West Indies, Trinidad and Tobago. Secondary data sources: Obtained through literature (Error! Not a valid result for table.)
Data accessibility	Direct URL to data: Chadee, Aron; Allis, Chamari; Rathnayake, Upaka (2023), “Data exploration on the factors associated with cost overrun on social housing projects in Trinidad and Tobago”, Mendeley Data, V3, doi:10.17632/46c7hr9r7v.3 Repository name: Mendeley Data Direct URL to data: https://data.mendeley.com/datasets/46c7hr9r7v/3
Related research article	Chadee, A. A., Martin, H., Chadee, X.T., Bahadoorsingh, S., & Olutoge, F. (2023). Root Cause of Cost Overrun Risks in Public Sector Social Housing Programs in SIDS: Fuzzy Synthetic Evaluation. Journal of Construction Engineering and Management, 149(11), 04023106. https://doi.org/10.1061/JCEMD4.COENG-13402

## 1 Value of the Data

• The data set is the first to provide a methodological classification of the leading root causes of cost overruns in public sector social development housing projects. This is useful in acquiring a deeper understanding of these leading root causes and validated against their theoretical ontologies.
• The data can be used in decision making research to show the uncertainty, imprecision and complexity of perceptions and heuristics used in the construction industry and their major influences on the economic viability of social developmental projects. The data set shifts the current research agenda in cost overrun studies, exposing the lack of attention to the true leading root causes of cost overruns and adds to contemporary academic debate by encouraging project and construction practitioners to reflect, refocus, reframe, and reset the research agenda to uncover key tacit knowledge areas.
• The data can be applied to develop forecasting models to demonstrate the misalignment in the construction housing industry and highlight the gaps in contemporary project practices leading to unsustainable delivery and practices of social housing. The data can be used as a basis of comparison with that of other Small Island Developing States and/or on a worldwide scale, in the field of construction project management. It further updates project management practices by uncovering and prioritising theoretical constructs critical to public sector projects.
• The provided data can be utilized by academia and construction project practitioners to develop a multitude of risk assessment processes, models and pragmatic tools based on these critical risk factors for further testing to optimize cost performances and sustainability on this value driven socially dependent infrastructure projects.
• The data can be used by policy makers and governmental bodies to analyse the latent effects of critical risk factors grouped under various root causes can have on overall developmental policies, and their emulation on the overall social housing value. These latent effects can be studied to develop strategies to mitigate wicked problems associated with social housing such as crime, unemployment, and income inequalities.

## 2 Data Description

The data was obtained through literature research, and structured questionnaires. A total of 150 questionnaires were distributed to Project Managers, Contractors, Engineers, Architects, and Consultants within the construction industry who have been involved in social housing projects [1]. The data received from the participants were presented as follows: The data on the highest level of education attained by the respondents is presented in Table 1 which illustrates that more than 70% of respondents have a minimum qualification of a Bachelor of Science degree, data on the professional role (Table 2) which highlighted that respondents represent mainly five professional roles, sector of employment in which they are employed (Table 3) either in the public sector or the private sector, types of projects mainly carried out by the organisations to which the participants belong (Table 4) under main eight categories, the number of employees (Table 5) where that most of the respondents are belonging to the organisations which are having more than 200 employees, number of projects participated in (Table 6), annual estimated turn over (Table 7), expected duration of projects (Table 8), and the actual time spent (Table 9). Table 10 presents data on the number of years of experience of each respondent in the field of project management, consultancy, contracting, engineering, and architecture. Data on the Risk Impact associated with cost overrun on construction projects compared between the private sector and public is presented in Table 11. The data clearly show that the impact of the factors that contribute to the cost overruns is different between the public and private sectors.

**Table 1:** Data highest level of education attained in the field of Civil engineering/ project management of the respondents.

Education	Frequency	Percent
Other (please specify)	6	4.0
BSc	89	59.3
MSc	29	19.3
MPhil	7	4.7
PhD	2	1.3
Diploma	17	11.3
Total	150	100.0

**Table 2:** Data on professional role in the company/organisation of the respondents.

Professional role	Frequency	Percentage
Project Manager	57	38
Contractor	13	8.7
Engineer	57	38
Architect	5	3.3
Consultant	20	13.3
Other	17	11.3

**Table 3:** Data of sector of employment of the respondents.

Employment sector	Frequency	Percent
Public	85	56.7
Private	65	43.3
Total	150	100.0

**Table 4:** Data on the nature of the organization's projects to which the respondents belong.

Nature of organisation	Frequency	Percentage
Transportation projects	22	14.7
Civil Engineering projects	92	61.3
Stadium/Exhibition/shopping center	33	22
Infrastructure Projects	81	54
Commercial projects	44	29.3
Water/Wastewater treatment project	16	10.7
Health service projects	20	13.3
Housing	75	50
Other	13	8.7

**Table 5:** Data of the number of employees in the organisation of the respondents.

Number of employees	Frequency	Percent
less than 20	23	15.3
21–50	28	18.7
51–100	22	14.7
101–150	14	9.3
151–200	4	2.7
more than 200	59	39.3
Total	150	100.0

**Table 6:** Data of the number of projects the respondents were involved in /participated in.

Number of projects	Frequency	Percent
0–5	55	36.7
6–10	33	22.0
11–15	16	10.7
16–20	9	6.0
more than 20	37	24.7
Total	150	100.0

**Table 7:** Data on the annual estimated turnover of the company in which the respondent is employed.

Turnover	Frequency	Percent
Less than 5 million USD	59	39.3
Over 5 million USD	91	60.7
Total	150	100.0

**Table 8:** Data of the expected duration estimated for the last executed project by the respondents.

Expected duration of the last project	Frequency	Percent
less than 12 months	48	32.0
12–18 months	39	26.0
18 months - 24 months	26	17.3
24–30 months	23	15.3
30–36 months	10	6.7
N/A	4	2.7
Total	150	100.0

**Table 9:** Data of the actual time spent to execute the last project by the respondents.

The actual duration of the last project	Frequency	Percent
less than 12 months	36	24.0
12–18 months	31	20.7
18–24 months	25	16.7
24–30 months	18	12.0
30–36 months	22	14.7
greater than 36 months	18	12.0
Total	150	100.0

**Table 10:** Data on the number of years of experience of the respondents.

Field	0–5 years	6–10 years	11–15 years	16–20 years	> 20 years	N/A years
Project Management	40%	20%	6%	10%	8%	16%
Consultancy	23.3%	10%	4.7%	1.3%	2%	58.7%
Contracting	20%	7.3%	6.7%	0.7%	6.7%	58.7%
Engineer	34.7%	12%	7.3%	10.7%	12.7%	22.7%
Architect	16%	1.3%	4%	——-	2%	76.7%

**Table 11:** Data on the level of risk associated with a cost overrun on construction projects.

Assessment statements	Private sector		Public sector	
	Risk impact	Overall rank	Risk impact	Overall rank
1. For public sector projects, cost overruns have become the ‘new normal’ or accepted culture.	5.64	4	5.36	8
2. Cost overruns undermine the viability of future projects.	5.42	7	5.60	5
3. Cost overruns can negatively impact taxpayers.	6.16	1	6.10	1
4. Psychological effects, such as optimism bias has a role in the cost overrun phenomena.	4.98	14	4.99	15
5. Project Actors are swayed by strategic misrepresentation, i.e. deception.	5.03	13	5.15	12
6. Politicians broker deals on construction projects.	5.52	6	5.59	6
7. Politicians lobbying for projects based on personal interest.	5.66	3	5.82	3
8. Project actors show a marked tendency to underestimate the duration of a project.	5.32	9	5.13	13
9. Project actors show a marked tendency to underestimate the cost of a project.	5.38	8	5.36	7
10. Project actors show a marked tendency to underestimate the risk associated with a project.	6.08	2	5.95	2
11. Construction projects often overrun the budget sum.	5.56	5	5.62	4
12. There are robust empirical data on substantial cost overruns.	4.49	17	4.55	17
13. Initial cost is underestimated.	5.24	10	5.32	9
14. Initial cost is unrealistic.	5.13	12	4.95	16
15. confidence in the government on project information is high.	4.83	16	5.18	11
16. Benefits to society are overestimated.	4.85	15	5.11	14
17. Value for money is less than anticipated.	5.16	11	5.31	10

Furthermore, Table 12 presented factors contributing to cost overrun on public sector projects which were extracted through the existing literature. The analysis of the raw data (factors presented in Table 12), provides the 22 critical factors associated with a cost overrun on public sector projects (Table 13) based on the severity and the probability of each risk whistle analysing the risk impact factor. The data in Table 14 (Data on the classification and ranking of critical risk factors), Table 15 (Data on the weightings for the 22 CRFs and 4 PRFs for Social Housing Program), Table 16 (Data on the membership function of all CRFs and PRFs for Risk Probability and Severity), Table 17 (Data on the membership function of the overall risk level), Table 18 (Data on the overall risk level) presents the levels to the fuzzy logic analysis approach implemented to rank the principal risk groups (Political, Socio-economical, technical and psychological) according to the risk index.

**Table 12:** Data on the 41 factors linked to cost overrun on public sector projects, were extracted and grouped through literature research.

**Table 13:** Data on the risk probability, severity, and risk impact along with the normalised values obtained for the risk factors associated with cost overrun.

ID	Risk Factors	Risk Probability		Risk Severity		RSI	Risk impact	Overall rank	Normalized values
		Mean	Rank	Mean	Rank				
32	Selection of politically aligned contractors	5.95	1	5.83	4	34.65	5.89	1	1.00
36	Contract Poorly designed (intentionally).	5.77	9	5.93	1	34.18	5.85	2	0.98
30	Project actors deliberately underestimate the cost to gain management approvals	5.79	7	5.86	2	33.91	5.82	3	0.97
33	Selection of political aligned project management team (i.e. consultant, team lead, directors etc.)	5.90	2	5.75	8	33.91	5.82	4	0.97
25	Strategic Misrepresentation i.e. lying e.g. underestimating costs.	5.76	10	5.84	3	33.64	5.80	5	0.96
28	Ministerial interference	5.86	3	5.74	9	33.64	5.80	6	0.96
31	Project actors deliberately overestimating the benefits of projects to society to justify viability.	5.83	5	5.75	7	33.56	5.79	7	0.95
19	lengthy bureaucratic processes	5.77	8	5.79	6	33.41	5.78	8	0.95
27	Pre-election commitments	5.85	4	5.67	10	33.13	5.76	9	0.93
29	Direct political influences (i.e. ministerial influences, location & type of project)	5.63	11	5.81	5	32.75	5.72	10	0.92
34	Political election cycles	5.80	6	5.31	15	30.82	5.55	11	0.83
26	Escalating commitment	5.56	12	5.53	13	30.77	5.55	12	0.83
1	Design change	5.32	14	5.58	11	29.69	5.45	13	0.78
9	Rework/Errors	5.19	16	5.25	16	27.28	5.22	14	0.67
35	Governance shortfall in the organization	5.21	15	5.23	17	27.25	5.22	15	0.66
6	Underestimation	4.61	29	5.55	12	25.59	5.06	16	0.58
20	Economic business cycles	5.03	17	4.97	21	25.00	5.00	17	0.55
21	Acquiring regulatory approvals	5.42	13	4.57	32	24.75	4.98	18	0.54
37	Optimism bias, i.e. judging future Project events in a positive light than the actual reality.	4.80	20	5.11	19	24.54	4.95	19	0.53
10	Technical uncertainty, i.e. poorly defined project objectives.	4.67	23	5.23	18	24.43	4.94	20	0.52
17	Labour strikes	4.57	32	5.34	14	24.39	4.94	21	0.52
24	Financial shortfalls	4.84	18	5.02	20	24.30	4.93	22	0.52
13	Schedule errors	4.81	19	4.87	25	23.39	4.84	23	0.47
38	Overcommitment/lock-in to a course of action	4.66	25	4.97	21	23.18	4.81	24	0.46
14	Legal implications	4.61	30	4.91	23	22.60	4.75	25	0.43
2	Client initiated variation	4.78	21	4.73	30	22.59	4.75	26	0.43
8	Scope change	4.67	23	4.83	26	22.59	4.75	27	0.43
16	Community involvement	4.63	27	4.77	28	22.09	4.70	28	0.40
23	Global financial crisis	4.59	31	4.78	27	21.92	4.68	29	0.39
18	Shortage of labour	4.63	28	4.53	33	20.97	4.58	30	0.34
15	Unknowns (e.g. earthquake)	4.35	35	4.76	29	20.69	4.55	31	0.33
40	Cognitive bias	4.72	22	4.38	35	20.67	4.55	32	0.33
11	Inexperience	4.65	26	4.23	37	19.70	4.44	33	0.27
39	Cautious attitude towards risks	4.37	34	4.47	34	19.53	4.42	34	0.26
5	The complexity of variables and conditions propagating design errors	4.05	39	4.66	31	18.89	4.35	35	0.22
22	Exploitation	3.84	40	4.91	23	18.84	4.34	36	0.22
4	Project procurement array of conditions	4.57	32	4.06	38	18.54	4.31	37	0.20
41	Prejudices	4.33	36	3.97	39	17.19	4.15	38	0.12
12	Innovation (novel) project	4.22	37	3.97	40	16.74	4.09	39	0.10
3	Sub-surface conditions	3.49	41	4.37	36	15.28	3.91	40	0.00
7	Omissions	4.11	38	3.71	41	15.22	3.90	41	0.00

^⁎⁎^RSI means Risk Significant Index= Probability x Severity; .$\text{Risk}\;\text{Impact}=\sqrt{RSI}$.

**Table 14:** Data on the classification and ranking of critical risk factors.

Critical risk factor (CRF) and category	Risk Impact	Overall ranking	Category ranking
***Political(CRG1):u_1_***			
Selection of politically aligned contractors, ***u*_11_**	5.89	1	1
Contract Poorly designed (intentionally), ***u*_12_**	5.85	2	2
Project actors deliberately underestimate the cost of gaining management approvals, ***u*_13_**	5.82	3	3
Selection of a politically aligned project management team (i.e. consultant, team lead, directors, etc.), ***u*_14_**	5.82	4	4
Strategic Misrepresentation i.e. lying e.g. underestimating costs., ***u*_15_**	5.80	5	5
Ministerial interference, ***u*_16_**	5.80	6	6
Project actors deliberately overestimating the benefits of projects to society to justify viability., ***u*_17_**	5.79	7	7
Pre-election commitments, ***u*_18_**	5.76	9	8
Direct political influences (i.e. ministerial influences, location & type of project), ***u*_19_**	5.72	10	9
Political election cycles, ***u*_110_**	5.55	11	10
Escalating commitment, ***u*_111_**	5.55	12	11
Governance shortfall in the organisation, ***u*_112_**	5.22	15	12
***Socio-Economical(CRG2):u*_2_**			
lengthy bureaucratic processes, ***u*_21_**	5.78	8	1
Economic business cycles, ***u*_22_**	5.00	17	2
Acquiring regulatory approvals, ***u*_22_**	4.98	18	3
Labour strikes, ***u*_24_**	4.94	21	4
Financial shortfalls, ***u*_25_**	4.93	22	5
***Technical(PRF3):u*_3_**			
Design change, ***u*_31_**	5.45	13	1
Rework/Errors, ***u*_32_**	5.22	14	2
Underestimation, ***u*_33_**	5.06	16	3
Technical uncertainty, i.e. poorly defined project objectives., ***u*_34_**	4.94	20	4
***Psychological(CRG4): u*_4_**			
Optimism bias, i.e. judging future project events in a positive light than the actual reality., ***u*_41_**	4.95	19	1

**Table 15:** Data on the weightings for the 22 CRFs and 4 CRGs for social housing program.

Critical Risk Factors (CRFs)	*Risk probability (p)*				*Risk severity (s)*			
	Mean probability	Weighing (*w_in_*) of CRF	Total mean of CRG	Weighting (*w_i_*) of CRG	Mean severity	Weighing (*w_in_*) of CRF	Total mean of CRG	Weighting (*w_i_*) of CRG
Selection of politically aligned contractors	5.95	0.086			5.83	0.085		
Contract Poorly designed (intentionally).	5.77	0.084			5.93	0.087		
Project actors deliberately underestimate the cost to gain management approvals	5.79	0.084			5.86	0.086		
Selection of political aligned project management team (i.e. consultant, team lead, directors etc.)	5.90	0.086			5.75	0.084		
Strategic Misrepresentation i.e. lying e.g. underestimating costs.	5.76	0.084			5.84	0.086		
Ministerial interference	5.86	0.085			5.74	0.084		
Project actors deliberately overestimating the benefits of projects to society to justify viability.	5.83	0.085			5.75	0.084		
Pre-election commitments	5.85	0.085			5.67	0.083		
Direct political influences (i.e. ministerial influences, location & type of project)	5.63	0.082			5.81	0.085		
Political election cycles	5.80	0.084			5.31	0.078		
Escalating commitment	5.56	0.081			5.53	0.081		
Governance shortfall in the organisation	5.21	0.076			5.2333	0.077		
***Political(PRF1):u_1_***			68.90	0.578			68.25	0.566
lengthy bureaucratic processes	5.77	0.225			5.79	0.225		
Economic business cycles	5.03	0.196			4.97	0.194		
Acquiring regulatory approvals	5.42	0.211			4.57	0.178		
Labour strikes	4.57	0.178			5.34	0.208		
Financial shortfalls	4.84	0.189			5.02	0.195		
***Socio-Economical(PRF2):u_2_***			25.63	0.215			25.69	0.213
Design change	5.32	0.269			5.58	0.258		
Rework/Errors	5.19	0.262			5.25	0.243		
Underestimation	4.61	0.233			5.55	0.257		
Technical uncertainty, i.e. poorly defined project objectives.	4.67	0.236			5.23	0.242		
***Technical(PRF3):u_3_***			19.80	0.166			21.61	0.179
Optimism bias, i.e. judging future project events in a positive light than the actual reality.	4.80	1.000			5.1133	1.000		
***Psychological(PRF4):***			4.80	0.040			5.11	0.042
Total of mean values of PRFs			119.13				120.66	

CRF = Critical Risk Factor, CRG= Critical Risk Group.

**Table 16:** Data on the membership function of all CRFs and CRGs for risk probability and severity.

Membership functions for all CRFs and PRFs for cost overrun on Social Housing Programs (Risk Probability)				Membership functions for all CRFs and PRFs for cost overrun on Social Housing Programs (Risk Severity)			
**CRFs&CRGs**	Weighing for CRFs	Membership function for level 3 (CRFs)	Membership function for level 2 (CRGs)	**CRFs&CRGs**	Weighing for CRFs	Membership function for level 3 (CRFs)	Membership function for level 2 (CRGs)
***Political(CRG1):***				***Political(CRG1):***			
Selection of politically aligned contractors	0.086	(0.01, 0.02, 0.01, 0.05, 0.15, 0.39, 0.36)	(0.012, 0.009, 0.035, 0.077, 0.194, 0.384, 0.290)	Selection of politically aligned contractors	0.085	(0.03, 0.01, 0.01, 0.09, 0.12, 0.39, 0.35)	(0.013, 0.011, 0.035, 0.093, 0.194, 0.374, 0.281)
Contract Poorly designed (intentionally).	0.084	(0.01, 0.02, 0.07, 0.06, 0.14, 0.33, 0.37)		Contract Poorly designed (intentionally).	0.087	(0.02, 0.01, 0.03, 0.05, 0.13, 0.36, 0.39)	
Project actors deliberately underestimating the cost to gain management approvals	0.084	(0.02, 0.00, 0.04, 0.08, 0.12, 0.45, 0.29)		Project actors deliberately underestimating the cost to gain management approvals	0.086	(0.01, 0.01, 0.02, 0.11, 0.12, 0.39, 0.35)	
Selection of political aligned project management team (i.e. consultant, team lead, directors etc.)	0.086	(0.01, 0.01, 0.02, 0.10, 0.12, 0.37, 0.37)		Selection of political aligned project management team (i.e. consultant, team lead, directors etc.)	0.084	(0.01, 0.02, 0.05, 0.07, 0.15, 0.37, 0.33)	
Strategic Misrepresentation i.e. lying e.g. underestimating costs.	0.084	(0.01, 0.01, 0.04, 0.08, 0.15, 0.43, 0.28)		Strategic Misrepresentation i.e. lying e.g. underestimating costs.	0.086	(0.01, 0.01, 0.03, 0.08, 0.18, 0.38, 0.32)	
Ministerial interference	0.085	(0.01, 0.01, 0.03, 0.06, 0.17, 0.37, 0.35)		Ministerial interference	0.084	(0.01, 0.02, 0.03, 0.08, 0.21, 0.35, 0.31)	
Project actors deliberately overestimate the benefits of projects to society to justify viability.	0.085	(0.01, 0.00, 0.03, 0.07, 0.17, 0.41, 0.30)		Project actors deliberately overestimating the benefits of projects to society to justify viability.	0.084	(0.01, 0.01, 0.03, 0.09, 0.12, 0.45, 0.28)	
Pre-election commitments	0.085	(0.01, 0.01, 0.03, 0.09, 0.17, 0.37, 0.33)		Pre-election commitments	0.083	(0.01, 0.01, 0.03, 0.13, 0.18, 0.35, 0.29)	
Direct political influences (i.e. ministerial influences, location & type of project)	0.082	(0.01, 0.01, 0.01, 0.03, 0.37, 0.32, 0.23)		Direct political influences (i.e. ministerial influences, location & type of project)	0.085	(0.01, 0.01, 0.03, 0.06, 0.17, 0.43, 0.29)	
Political election cycles	0.084	(0.01, 0.01, 0.02, 0.05, 0.19, 0.46, 0.25)		Political election cycles	0.078	(0.01, 0.01, 0.02, 0.09, 0.43, 0.35, 0.09)	
Escalating commitment	0.081	(0.01, 0.00, 0.08, 0.15, 0.14, 0.30, 0.31)		Escalating commitment	0.081	(0.01, 0.00, 0.09, 0.15, 0.17, 0.27, 0.32)	
Governance shortfall in the organization	0.076	(0.01, 0.01, 0.04, 0.09, 0.43, 0.39, 0.03)		Governance shortfall in the organisation	0.077	(0.01, 0.01, 0.04, 0.11, 0.37, 0.41, 0.04)	
***Socio-Economical(CRG2):***				***Socio-Economical(CRG2):***			
lengthy bureaucratic processes	0.225	(0.01, 0.01, 0.03, 0.13, 0.11, 0.40, 0.31)	(0.015, 0.023, 0.053, 0.180, 0.314, 0.313, 0.103)	lengthy bureaucratic processes	0.225	(0.01, 0.03, 0.05, 0.07, 0.15, 0.36, 0.35)	(0.009, 0.024, 0.068, 0.187, 0.291, 0.273, 0.148)
Economic business cycles	0.196	(0.01, 0.01, 0.04, 0.17, 0.45, 0.31, 0.02)		Economic business cycles	0.194	(0.01, 0.01, 0.10, 0.14, 0.38, 0.34, 0.02)	
Acquiring regulatory approvals	0.211	(0.01, 0.01, 0.05, 0.11, 0.29, 0.34, 0.19)		Acquiring regulatory approvals	0.178	(0.01, 0.03, 0.07, 0.36, 0.41, 0.09, 0.04)	
Labour strikes	0.178	(0.03, 0.06, 0.13, 0.13, 0.38, 0.27, 0.00)		Labour strikes	0.208	(0.01, 0.05, 0.09, 0.14, 0.15, 0.31, 0.25)	
Financial shortfalls	0.189	(0.01, 0.02, 0.01, 0.36, 0.33, 0.26, 0.02)		Financial shortfalls	0.195	(0.02, 0.00, 0.04, 0.22, 0.38, 0.28, 0.06)	
***Technical(CRG3):***				***Technical(CRG3):***			
Design change	0.269	(0.03, 0.00, 0.01, 0.07, 0.44, 0.39, 0.06)	(0.020, 0.00, 0.023, 0.236, 0.457, 0.211, 0.052)	Design change	0.258	(0.01, 0.02, 0.01, 0.06, 0.39, 0.30, 0.22)	(0.007, 0.015, 0.015, 0.081, 0.437, 0.305, 0.140)
Rework/Errors	0.262	(0.02, 0.00, 0.01, 0.10, 0.53, 0.28, 0.06)		Rework/Errors	0.243	(0.01, 0.01, 0.02, 0.07, 0.52, 0.33, 0.04)	
Underestimation	0.233	(0.01, 0.00, 0.04, 0.41, 0.44, 0.05, 0.05)		Underestimation	0.257	(0.01, 0.02, 0.01, 0.09, 0.39, 0.23, 0.25)	
Technical uncertainty, i.e. poorly defined project objectives.	0.236	(0.02, 0.00, 0.03, 0.37, 0.43, 0.12, 0.03)		Technical uncertainty, i.e. poorly defined project objectives.	0.242	(0.01, 0.01, 0.03, 0.10, 0.45, 0.35, 0.05)	
***Psychological(CRG4):***				***Psychological(CRG4):***			
Optimism bias, i.e. judging future project events in a positive light than the actual reality.	1.000	(0.01, 0.01, 0.04, 0.28, 0.49, 0.12, 0.05)	(0.01, 0.01, 0.04, 0.28, 0.49, 0.12, 0.05)	Optimism bias, i.e. judging future project events in a positive light than the actual reality.	1.000	(0.02, 0.01, 0.05, 0.10, 0.44, 0.33, 0.05)	(0.02, 0.01, 0.05, 0.10, 0.44, 0.33, 0.05)

CRF = Critical Risk Factor, CRG= Critical Risk Group.

**Table 17:** Data on the membership function of the overall risk level (level 1).

Critical risk groups (CRGs)	Weighing for CRGs	Membership function of level 2 (CRGs)	Membership functions of level 1 (ORL)
Risk Probability:			
***Political(CRG1):***	0.578	(0.012, 0.009, 0.035, 0.077, 0.194, 0.384, 0.290)	(0.014, 0.010, 0.037, 0.134, 0.275,0.330, 0.200)
***Socio-Economical(CRG2):***	0.215	(0.015, 0.023, 0.053, 0.180, 0.314, 0.313, 0.103)	
***Technical(CRG3):***	0.166	(0.020, 0, 0.023, 0.236, 0.457, 0.211, 0.052)	
***Psychological(CRG4):***	0.040	(0.01, 0.01, 0.04, 0.28, 0.49, 0.12, 0.05)	
Risk Severity:			
***Political(CRG1):***	0.566	(0.013, 0.011, 0.035, 0.093, 0.194, 0.374, 0.281)	(0.011, 0.015, 0.039, 0.111, 0.269, 0.339, 0.217)
***Socio-Economical(CRG2):***	0.213	(0.009, 0.024, 0.068, 0.187, 0.291, 0.273, 0.148)	
***Technical(CRG3):***	0.179	(0.007, 0.015, 0.015, 0.081, 0.437, 0.305, 0.140)	
***Psychological(CRG4):***	0.042	(0.02, 0.01, 0.05, 0.10, 0.44, 0.33, 0.05)	

CRG= Critical Risk Group.

**Table 18:** Data on the Overall Risk Level (ORL).

Critical risk group (PRFs)	Probability of Occurrence		Severity		Overall Risk level		Ranking
	Index	Linguistic	Index	Linguistic	Index	Linguistic	
***Political(PRF1):***	5.743	Very High	5.690	Very High	5.716	Very High	1
***Socio-Economical(PRF2):***	5.104	High	5.138	High	5.121	High	2
***Technical(PRF3):***	4.953	High	5.403	High	5.173	High	3
***Psychological(PRF4):***	4.800	High	5.113	High	4.954	High	4
***OverallRiskLevel(ORL)***	5.437	High	5.496	High	5.466	High	–

## 3 Experimental Design, Materials and Methods

The population of the data consists of professionals within the construction industry namely Project Managers, Contractors, Engineers, Architects, and Consultants. A total of 150 questionnaires were distributed, 84 via the online SurveyMonkey tool. These included 15 email invitations with 5 responses (33.33%) and 79 web links. Additionally, 66 hard-copy questionnaires were administered and returned. Evidence from the literature provided 41 risk factors associated with cost overruns on public sector projects within the construction industry. The responses were rated on a 7-point Likert scale (1 = extremely low, 2 = very low, 3 = low, 4 = moderate, 5 = high, 6 = very high, 7 = extremely high), to determine the probability and severity of each risk factor. The data collected was analysed in the Statistical Package for Social Sciences (SPSS) IBM 25. Descriptive statistical tools such as frequency, percentage, and mean were used to present the data. Calculations of the risk impact (RI) values, normalisation values, and ranking were carried out in Microsoft Excel 2018.

The respondents’ perceptions of the problematic issues related to cost overrun on public sector infrastructure development projects (PSIDPs) were ranked distinctly according to the sector of employment of the respondents (Table 11).

The 22 critical factors contributing to cost overrun within Trinidad and Tobago public sector projects were obtained through normalisation of the risk impact (RI) values of the 41 factors and ranked according to the normalised values obtained so that factors having values greater than 0.5 were deemed critical (Table 13).

Through the application of fuzzy logic, namely fuzzy synthetic evaluation, the 22 critical risk factors (CRFs) were classified under four critical risk groups (CRGs), namely, political, socio-economical, technical, and psychological, and ranked overall according to their category, based on the risk impact (Table 14) [1]. The weighing function, of the CRFs, (second-level) and CRGs (first level) are calculated from the mean values, obtained through SPSS for both its probability and severity (Table 15). Next, the membership functions of the CRFs & CRGs (level 1) along with the risk level of each CRG (MF level 2) were determined and presented in Table 16. The obtained fuzzy evaluation matrixes, $D_{i}\left(i=u_{1},\;u_{2},\;u_{3},\;u_{4}\right)$ of the CRGs (level 2) were further normalized by considering their weighing functions to generate the final fuzzy evaluation matrix of overall risk level (ORL) of cost overrun of social housing development (i.e. level 1). The probability and severity matrixes of the PRFs are represented in column 3 of Table 17. The overall risk level of cost overrun on public sector projects in developing countries is presented in Table 18 which illustrates that the political category has more risk compared to the others.

The outcome of this study indicates that further studies could be conducted to evaluate the cost controlling and monitoring strategies for the identified risk factors of cost overrun on social housing projects and a study on cost planning and estimating mechanisms to mitigate the factors of cost overrun on social housing projects could also be carried out. Furthermore, similar types of studies can be conducted for the other types of building and infrastructure construction projects which will contribute greatly to the existing knowledge and the betterment of the industry.

## Limitations

None.

## Ethics Statement

The proposed data does not involve any human subjects, animal experiments, or data collected from social media platforms.

## CRediT authorship contribution statement

**Aaron Anil Chadee:** Conceptualization, Methodology, Software, Writing – original draft, Investigation. **Chamari Allis:** Validation, Writing – review & editing. **Upaka Rathnayake:** Validation, Writing – review & editing. **Hector Martin:** Validation, Writing – review & editing. **Hazi M Azamathulla:** Validation, Writing – review & editing.

## Data Availability

Data exploration on the factors associated with cost overrun on social housing projects in Trinidad and Tobago (Original data) (Mendeley Data)
